# The Giant Condyloma (Buschke-Löwenstein Tumor) in the Immunocompromised Patient

**DOI:** 10.1155/2014/793534

**Published:** 2014-09-25

**Authors:** Andrew L. Atkinson, Nicole Pursell, Abinet Sisay

**Affiliations:** ^1^Department of Obstetrics and Gynecology, Jersey Shore University Medical Center, 1945 Route 33, Neptune, NJ 07753, USA; ^2^Department of Obstetrics and Gynecology, Debre Markos Referral Hospital, Debre Markos, Ethiopia

## Abstract

Since Buschke and Löwenstein first described the giant condyloma in 1925 (which subsequently was named Buschke-Löwenstein tumor), there have been scattered reports over the past 90 years describing presentation and different avenues of treatment for patients with this condition. It is well known that immunocompromised individuals are at an increased risk of anogenital disease caused by human papillomavirus (HPV). In this report, we present the management of two HIV positive patients with giant condylomas. Both patients presented with urinary outflow obstruction and sepsis. Though giant condylomas are a rare phenomenon, these two cases underscore the importance of early treatment intervention, especially in the immunocompromised patient.

## 1. Introduction

The giant condyloma was first described in a male patient in 1925 by Buschke and Löwenstein [1]. Giant condylomas are an exceedingly rare condition with an estimated incidence of 0.1% in the general population, with its pathogenesis and natural history not well understood [2–5]. The ratio between men and women is 3 : 1, and the mean age of occurrence is approximately 50 years [6]. Human papillomavirus (HPV) types 6 and/or 11 DNA is regularly found in giant condylomas, strongly suggesting a pathogenic role in tumor development [7]. The immune system effectively wards off the majority of HPV infections in the healthy individual and is associated with marked localized cell mediated immune responses [8]. However, in patients that are immunocompromised, there is an increased risk of developing vulvar, vaginal, or perianal lesions associated with HPV [8–10]. Patients with high susceptibility to local development, fast progression (growth and malignancy), and high rates of recurrence often exhibit various types of immunodeficiency [11]. In addition, immunodeficiency leads to difficulties in optimal therapeutic management and outcomes.

We report two cases of giant condylomas found in patients in northern Ethiopia who presented to a referral hospital for medical treatment. Both patients at the time of presentation were septic and would have inevitably died from their condition if left untreated. This report outlines the dangers of the giant condyloma in the immunocompromised female patient and the possible sequelae that can arise if left untreated.

## 2. Case Presentation

**Figure 1:**
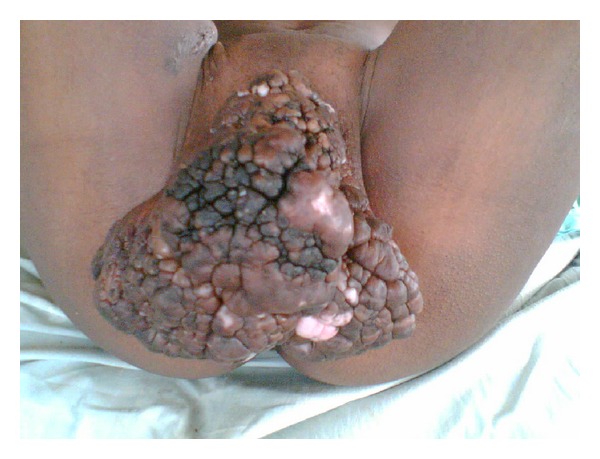
Patient “A” placed in dorsal lithotomy position revealing giant condyloma completely covering vulva.

Patient “A” was a 29-year-old nulligravid HIV positive female who presented to Debre Markos Referral Hospital accompanied by her mother who stated that her daughter had been acting “strange” for the past couple of days. Upon initial presentation it was noted that the patient was lethargic, diaphoretic, and tachypneic. Her vitals were a blood pressure of 82/49 mmHg, heart rate 129, respiratory rate 42, pulse oximetry 79%, and a temperature of 39.3 Celsius. On physical exam it was noted that the patient had a giant condyloma that completely engulfed her external urethral orifice, vestibule, as well as the vaginal introitus (Figure 1). A transurethral catheter was placed which produced 1200 cc's of dark tea colored, foul smelling urine. To place the urinary catheter, the patient was placed in lithotomy position; the mass was then elevated to reveal the vaginal introitus and then finally the urethral orifice. The mass grew from the labia majora bilaterally and also had attachments to the mons pubis anteriorly; there was no growth in the posterior region. It is worthwhile to note that if the mass was not mobile enough to reach the urethra or if hemorrhage occurred with manipulation, a suprapubic catheter would have been considered.

Standard labs were drawn along with blood and urine cultures. The patient was started on ceftriaxone as well as 5% dextrose in normal (0.9%) saline (D5NS) for fluid volume resuscitation. Laboratory results were remarkable for a white blood cell (WBC) count of 29,100 cells/mcl and creatinine of 7.2 mg/dL, and both blood and urine cultures grew* Escherichia coli* (*E. coli*). A CD4 count was 250 cells/*μ*L, with a viral load >100,000 copies/mL. Because of the location and lack of resources of this particular facility in Ethiopia, neither HPV genotyping nor histological examination to rule out malignancy could be performed. Also, ideally a CT scan or MRI would be performed prior to surgery to detail depth of invasion as well as subcutaneous spread, but again because of the remote location these modern luxuries were not available.

Fluid resuscitation was guided by blood pressure, heart rate, and urine output. The patient was maintained on D5NS @ 150 cc/hr, with boluses of fluid using normal saline being given as needed. Urine output for the first 48 hours was less than 250 cc's. On hospital day #3, the patient's urine output over 24 hrs was approximately 800 cc's, and a repeat creatinine was 3.2 with the value trending down daily until it normalized by hospital day #5. The patient's renal failure also caused hyperkalemia, with the potassium value being 6.2 mEq/L on admission. Dialysis was not an option; hyperkalemia was treated with insulin therapy in conjunction with dextrose in the intravenous fluids. After 5 days of aggressive fluid hydration and intravenous antibiotic treatment, the patient's status stabilized with electrolytes, renal status, and WBC count returning to normal parameters. The patient consented for surgical intervention and was taken to the operating theatre.

**Figure 2:**
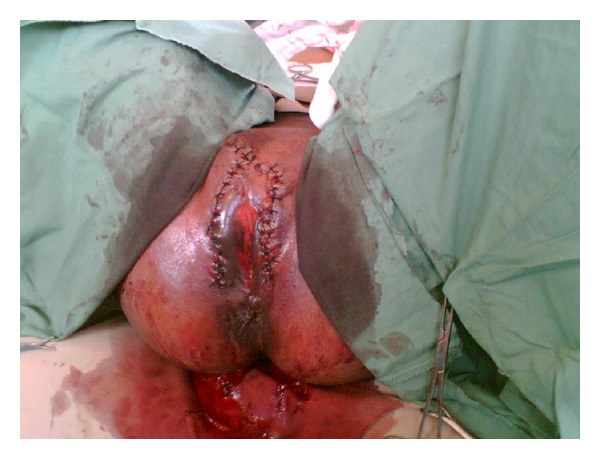
The giant condyloma completely excised along with the labia majora. Incision was closed with interrupted suture.

The patient was placed in dorsal lithotomy position, prepped, and draped. The urinary catheter was left in place, and the voluminous mass of condyloma was handled with a sterile towel. The mass was shifted to the patient's right side to reveal the left aspect of the vulva. On inspection it was found that the majority of the condyloma was growing from the labia majora. A decision was made to excise the labia majora freeing the mass from the body. The excision site extended anteriorly from the dorsal aspect of the left labia majora to the mons pubis and continued posteriorly along the right labia majora, in the shape of an inverted “U” (Figure 2). The invasion of the condyloma was estimated at time of surgery to be less than a centimeter, which is remarkable considering the size and weight of the mass. The patient tolerated the procedure well; total blood loss was estimated at 300 cc's, and the total weight of the excised condyloma was 1875 grams.

Patient “A” made a full recovery and was discharged from the hospital on postoperative day #7. The patient was given instructions to keep the area clean with soap and water while keeping the area as dry as possible covering it with clean underwear daily. The patient was also discharged with a ten-day course of cephalexin for prophylaxis against wound infection as well as HAART therapy for her HIV. She returned to the hospital on postoperative day #14 for suture removal as well as for wound surveillance. The vulva was healing well with no signs of wound breakdown or infection. She was followed up at 6-months for an inspection for recurrence in which there was none; a repeat viral load at that time was also nondetectable. The patient was in good spirits. She had previously been living in isolation, ashamed of her condition, and after surgical intervention, she stated that she was slowly integrating herself back into village society.

**Figure 3:**
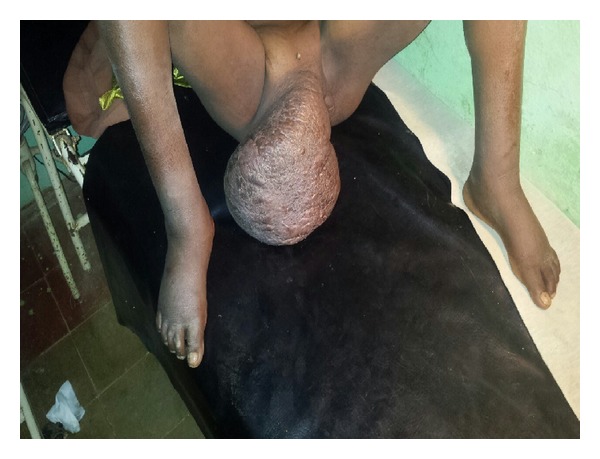
Patient “B” on initial presentation. Giant condyloma noted to be growing off right aspect of vulva with extension to the mons pubis.

Patient “B” was a 24-year-old nulligravid HIV positive female who presented to Debre Markos Hospital accompanied by her mother and sister. The patient's chief complaint upon presentation was abdominal pain and fevers. The patient had a blood pressure of 92/63 mmHg, heart rate 111, respiratory rate 28, pulse oximetry 92%, and a temperature of 38.6 Celsius. On physical exam it was noted that the patient had a giant condyloma that grew from the right side of the vulva and mons pubis, completely sparing the left aspect of the vulva (Figure 3). When a transurethral catheter was placed, 300 cc's of foul smelling dark urine was drained. Again, the patient was placed in dorsal lithotomy position for catheter placement. The mass was manipulated to the patient's right side to reveal the urethra and the catheter was placed without difficulty.

Routine labs and cultures were drawn. The patient's WBC count was 18,760 cells/mcl, creatinine 2.3 mg/dL, potassium 5.5 mEq/L, and the urine culture grew* E. coli* with the blood culture being negative. A CD4 count was 190 cells/*μ*L, with a viral load >100,000 copies/mL. The patient was started on ceftriaxone and D5NS @ 150 cc/hr with normal saline being used for boluses as needed. After three days of resuscitation the patient's vitals and labs stabilized.

**Figure 4:**
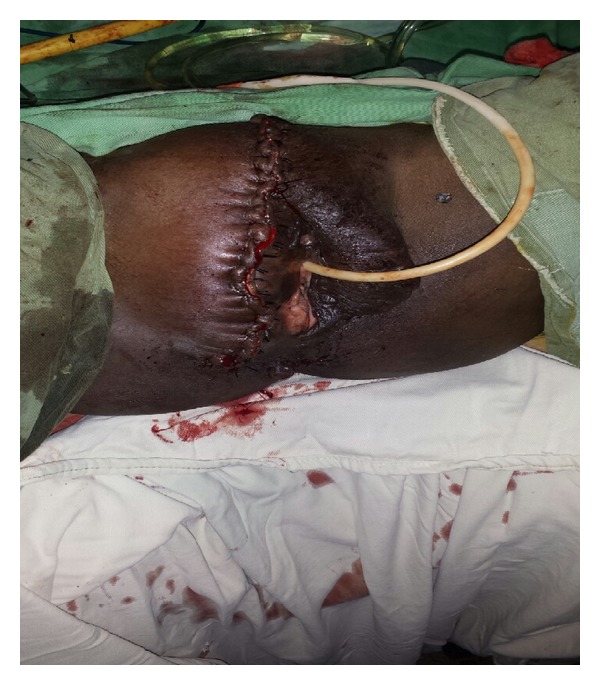
Giant condyloma excised from patient “B.” The right labia majora was excised with the specimen with minimal invasion noted. The left side of the vulva was spared.

The patient was taken to the operating theatre where she was placed in dorsal lithotomy position and draped. Once again it was noted that the condyloma was growing from the labia majora with an extension to the mons. Unlike patient “A,” this condyloma did not cross the midline. The condyloma was excised and the incision site was closed with interrupted suture (Figure 4). Again it was noted that the condylomas depth of invasion at the time of surgery was less than a centimeter. Total blood loss for the case was 200 cc's, and the weight of the condyloma was approximately 2010 grams.

Patient “B” was discharged from the hospital on postoperative day #9. Prior to discharge she was started on HAART therapy for her HIV as well as a ten-day course of cephalexin for prophylaxis against wound infection. She was followed up on postoperative day #20 for suture removal and wound surveillance. It was noted that there was a 3 cm wound separation on the posterior aspect of the incision site. There was no sign of infection, the old sutures were all removed, and the area of wound separation was irrigated and cleansed with betadine. Two mattress sutures were placed and the patient was told to return in 10 days for reexamination. Ten days later the patient returned and the sutures were removed, the skin came together nicely, and once again there were no signs of infection or dehiscence. She was seen 6 months later to check for recurrence. She had small perianal condylomas that were easily excised at the bedside, but no significant recurrence was noted. Her viral load at her 6-month follow-up was also nondetectable.

## 3. Discussion

These are the first described cases of complete urethral obstruction secondary to condylomata disease resulting in renal failure and urosepsis in two patients. Once the patients were stabilized, given the extent of the disease and the limited resources in the region, the most appropriate approach to management was surgical resection. There are reports of giant condylomas treated successfully with podofilox cream, topical 5-fluorouracil, regional radiation, CO2 laser, or a combined chemoradiation regimen [11–15]. Surgical excision is still the first line treatment for the giant condyloma though, with higher success rates (53–91%) and lower rates of recurrence [16]. In the third world setting, other options as mentioned above are not easily accessible and have not proven to have superior outcomes to surgical management. For example, it has been found that cure rate for the patient with radical surgical excision is 61% versus 25% in patients who underwent chemoradiation for treatment of the giant condyloma [17].

In the ideal setting, histological evaluation is preferred for these cases because in the immunocompromised patient, giant condylomas favor rapid growth and increased risk of malignant transformation, potentially favoring the oncogenetic mechanisms caused by HPV infection [17]. It is estimated that 56% of giant condylomas have malignant transformation, but interestingly there has never been a reported case of distant metastases [17]. There is an estimated mortality rate in patients with giant condylomas of approximately 20%, but this is hard to justify when the incidence of the disease itself is extremely rare and most published data on giant condylomas are scattered case reports over the past 90 years [6].

Recurrence is a big problem with giant condylomas particularly in the immunocompromised patient. It is estimated that the recurrence rate is as high as 66% [17]. The two patients presented in this report were followed up at one week, 6 months, and one year. It is our recommendation to see patients with a history of giant condyloma every 6 months in the first two years after surgery and then annually. The average time period of recurrence is approximately 10 months [17]. It is also important to start the patient on HAART therapy as soon as possible if their HIV is not adequately controlled. In the third world setting, a full physical exam should always be performed each time the immunocompromised patient make makes an office visit. To diagnose a giant condyloma in the early stages reduces both medical and surgical morbidity as well as overall mortality.

In conclusion, we present two cases of giant condyloma in a rural African setting that was complicated by urinary outflow obstruction leading to urosepsis. Medical stabilization followed by surgical excision is the treatment of choice in the immunocompromised patient especially in the third world setting. Starting the patient on HAART therapy as well as close follow-up in the first two years after surgery is necessary to manage recurrence and to decrease further morbidity and mortality from this condition.

The global impact of the Buschke-Löwenstein tumor on a patient is tremendous, and in both cases, the women affected were hidden from the public eye with the assistance of their families in shame and confusion. Furthermore, they had to adapt to walk, sit, and sleep with the large mass between their legs. Not only was the giant condyloma a physical burden, but also the mental and social toll that transpired was in some ways much more detrimental. Adequate education to immunocompromised individuals as well as to their families about what to look for on their bodies and when to seek out medical help would only take a few moments of a practitioner's time and could help save someone a lifetime of needless shame.
